# Study protocol of a randomized controlled trial to improve cancer prevention behaviors in adolescents and adults using a web-based intervention supplemented with SMS

**DOI:** 10.1186/1471-2458-13-357

**Published:** 2013-04-17

**Authors:** Alberto Lana, Maria Olivo del Valle, Santiago López, Goretti Faya-Ornia, Maria Luisa López

**Affiliations:** 1 University Institute of Oncology of Asturias, Central University Hospital of Asturias, C/Emilio Rodríguez Vigil s/n, 33006 Oviedo, Spain; 2 Department of Preventive Medicine and Public Health, Faculty of Medicine and Health Sciences, University of Oviedo, Avda. Julián Clavería s/n, 33006 Oviedo, Spain; 3 Department of Nursing, University of Cantabria, Faculty of Nursing, Avda. de Valdecilla s/n, 39008 Santander, Spain; 4 Department of Anglo-German and French Philology, University of Oviedo, Faculty of Philosophy and Letters, C/Teniente Alfonso Martínez s/n, 33011 Oviedo, Spain

**Keywords:** Adolescent, Randomized controlled trial, Internet, Cellular phone, Health behavior, Behavior control/methods, Health education, Prevention and control, Neoplasms/prevention and control, Models, Psychological

## Abstract

**Background:**

The overall number of cancer cases is increasing and, therefore, strengthening cancer prevention has become a priority. The institutions responsible for its control establish guidelines for primary prevention. These include recommendations, such as: not smoking, following a healthy diet, doing daily physical exercise or avoiding overweight. Adolescence is a period of adoption and/or consolidation of health behaviors, and both school- and family-based interventions have proven effective to improve them. Furthermore, online and mobile phone educational interventions are encouraging. Consequently, the main aim of this study is to assess the efficacy of an intervention in which these requirements (school, family, the Internet and SMS) are combined to prevent behavioral cancer risk.

**Methods:**

This protocol describes the design and implementation of a complex online program that includes a randomized controlled trial put into practice in two countries: Spain and Mexico. Adolescents and adults of their environment (relatives and teachers) who voluntarily participate will be randomly assigned to the experimental group or to the control group once they have completed the online pre-test. The experimental group members will have free access to a tailor-made and interactive website (http://www.alertagrumete.com). During the academic year, this website will be periodically updated with different school and leisure activities related to the avoidance of risk behaviors. To encourage participation, the program includes a competition that gives rewards to the winners. SMS are also sent to students to stimulate the adoption of healthy behaviors and as a reminder of participation. Finished the intervention, an online post-test is performed in both groups and the impact on the risk behaviors is therefore assessed.

**Discussion:**

The program is pioneer, since it combines many components which have already proven effective in previous researches. Moreover, it aims to compare efficacy in two countries with different socio-economic levels to find out if these approaches are equally effective in countries with a lower income level. However, the vertiginous evolution of the Internet and mobile phones may make this tool less attractive for adolescents, who may prefer social networks and other mobile phone applications which are nowadays massively used by their peers.

**Trial registration:**

ISRCTN27988779

## Background

### The current situation of cancer

In spite of the numerous implemented strategies for cancer control, it has not yet been possible to slow down the increasing number of diagnosis of this disease around the world. According to different estimates, in the less economically developed countries the incidence of cancer cases will be multiplied during the next decades; even in the richest countries, an immediate decrease is not expected [1]. If we also bear in mind the significant consequences (both mortal and non-mortal) that frequently take place after cancer is diagnosed, we could conclude that it is the disease which people and governments are most worried about [2]. Consequently, the percentage of the total health expenditure which is invested in cancer has increased in all countries, and it could even be unsustainable in the near future. For this reason, nowadays prestigious institutions are demanding an urgent improvement and the implementation of prevention strategies [3].

Although the main reason that underlies the relative failure in cancer control is the aging of the population, the external risk factors are the ones that lead to accumulative damages in the genome, and which end up causing the disease [4]. Therefore, we consider it is essential to suppress all the modifiable risk factors that are basically associated to health-related behaviors. According to the most current estimates, around 50% of the cancer cases per year could be avoided if the main triggering behaviors were suppressed [5-7].

### Guides for prevention

Different institutions of virtually all regions of the world establish and periodically broadcast advice about cancer prevention [8,9]. These recommendations seem to be effective in the reduction of (a) the global risk for cancer – as stated by the EPIC study [10], which is based in the assessment of more than 386,355 participants in nine European countries – and of (b) other chronic diseases of high prevalence [11]. One of the easiest and most complete guides is the “European Code Against Cancer” (ECAC) [12], which points out that “many aspects of general health can be improved and many cancer deaths prevented, if we adopt healthier lifestyles” (Table 1). The fourth revision of this guide is currently under elaboration.

**Table 1:** **European Code Against Cancer** (**third version**): **primary prevention advice**

**Risk behaviors**	**Advices**
Smoking	1. Do not smoke; if you smoke, stop doing so. If you fail to stop, do not smoke in the presence of non-smokers.
Overweight	2. Avoid obesity.
Sedentary	3. Undertake some brisk, physical activity every day.
Unhealthy diet	4. Increase your daily intake and variety of vegetables and fruits: eat at least five servings daily. Limit your intake of foods containing fats from animal sources.
Alcohol consumption	5. If you drink alcohol, whether beer, wine or spirits, moderate your consumption to two drinks per day if you are a man or one drink per day if you are a woman.
Ultraviolet radiation exposure	6. Care must be taken to avoid excessive sun exposure. It is specifically important to protect children and adolescents. For individuals who have a tendency to burn in the sun, active protective measures must be taken throughout life.
Workplace exposure to carcinogens	7. Apply strictly regulations aimed at preventing any exposure to known cancer-causing substances. Follow all health and safety instructions on substances which may cause cancer. Follow advice of national radiation protection offices.

### The current situation of primary prevention of cancer

During the last years, different intervention programs have been created with the aim of modifying, individually or collectively, the risk behaviors that refer to the above mentioned code. Some of them have had great success and others less, but what is sure is that all of them have contributed to identify those aspects that could increase the efficacy of this kind of programs. In these studies, we can observe that all experts agree on highlighting the importance of acting first and foremost on groups with a high risk. We consider the membership in these groups should preferably be based on socioeconomic or behavioral criteria [13], since the selection according to genetic predisposition could cause inequalities because the use of this resource is not generalized [14]. In addition we should bear in mind that children and adolescents are a particularly vulnerable group, as it is at this crucial stage when the main cancer risk behaviors start and are consolidated. Furthermore, scientific literature also warns that the preventive interventions should not be dissociated from the environment in which these behaviors are usually acquired (in the case of adolescents, school and family environment) [15,16]. In this respect, the Internet is a tool that could act as a link with the environments that are frequently visited by young people and, in addition, it perfectly adapts to their linguistic and behavioral codes. Related to this idea, Albreht et al. [17] point out that in order to face the challenges that an integral fight against cancer involves, research should be focused on those interventions that bring us closer to the social marketing. In the case of adolescents, it inevitably leads to the Internet. There are different examples of web-based interventions which are able to modify risk behaviors [18-21], and there are even some manuscripts which describe protocols of similar studies [22-24]. However, since this is a very recent approach, there is not yet an absolute consensus about its utility [25], and therefore further research in this field is still needed.

In addition, it is also well-known that multiple interventions are increasingly necessary, and that is because (a) individuals usually have more than only one risk behavior, and mainly since (b) this kind of interventions require fewer resources.

To conclude, we would like to highlight that the most promising strategies should be based on others that have proven effective, and above all, on psychosocial theories, which are the ones that best explain the behaviors we wish to prevent [26].

### Aim

This article describes the design of a program called “Prevencanadol” (acronym for the words Prevention/Cancer/Adolescents). Its main aim is to assess the impact of an educational intervention managed via the Internet and mobile phones in order to prevent the behavioral cancer risk among adolescents and their adult environment (family and school). Its secondary aims are: (a) to evaluate the feasibility of the program and (b) to identify the educational needs of focal groups and their priority.

## Methods and design

### Type of study

A randomized controlled trial (using pre- and post-intervention assessments) has been designed in order to determine the impact that this web- and SMS-based intervention has on the Spanish and Mexican population between 12 and 16 years old and their adult environment regarding the following of the ECAC primary prevention advice. The design also includes a study of feasibility (i.e., assessment of the implementation procedure), as well as a descriptive study of the risk behaviors showed by the participants of the pre-test, in order to identify those individuals with the highest behavioral cancer risk.

### Study population and composition of the groups

The study population is composed of Spanish and Mexican students of Secondary Education, aged between 12 and 16, who have voluntarily accepted to participate in an educational program which is managed online and supplemented with the delivery of SMS. Due to the program characteristics, it is required to have access to the Internet at home or at school. However, mobile phones are optional. Adults of the environment of the student (family or teachers) can also participate in the program: (a) they can be previously invited by an adolescent or (b) they can also join spontaneously.

Students and adults should register onto the Prevencanadol website. Then, a computerized and randomized process will distribute them in an experimental group (EG) and in a control group (CG). According to the program characteristics, the randomization is subject to these two requirements: (a) 60% of the users should be assigned to the CG since a higher withdrawal rate is expected in this group; and (b) members of the same classroom or family should share a study group so as to avoid the possible contamination of the groups.

In order to detect a significant decrease (p<0.05) of at least 10 percentage points in the global behavioral risk indicator during the post-test, it will be necessary to recruit 622 individuals in each arm of the study (statistical power = 90% and estimated withdrawals = 20%).

### Theoretical frame

**Figure 1:**
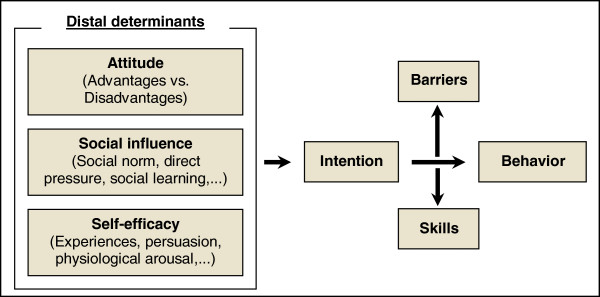
Brief graphic explanation of the A.S.E. Model.

As previously mentioned, spreading the ECAC advice and trying to change behaviors using the Internet to avoid behavioral cancer risk are already justified aims. However, this initiative could possibly fail if we do not bear in mind a psychological model that explains the possible risk behaviors. On the one hand, we should mention the A.S.E. Model (or I-Change Model), which was developed by de Vries group [27]. This model affirms that the purpose of carrying out a behavior mainly depends on three determining factors: attitude, social influence and self-efficacy (Figure 1). On the other hand, another well-known model among researchers is the Transtheoretical Model of Behavior Change, which was created by Prochaska and DiClemente [28]. This points out that the shifting from one risk behavior to a healthy one involves several stages of change. Both models have been used for the design of the educational tool.

### Educational intervention

The main tool for the implementation of the intervention is a website designed by experts in the field of Health Education, and which has been adapted for Spain (http://www.alertagrumete.com) and Mexico (http://www.alertagrumete.com.mx).

**Figure 2:**
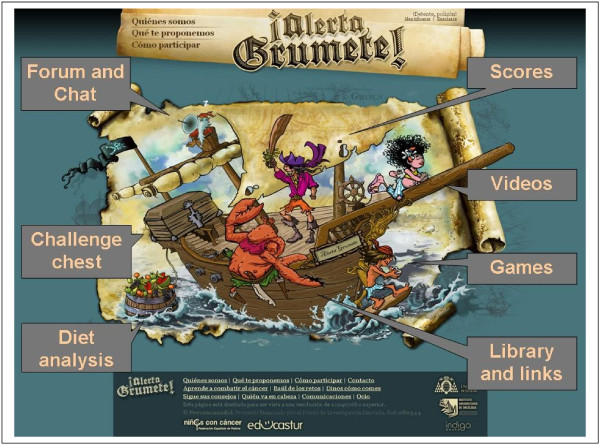
Home page and sections of Prevencanadol program website.

In a previous stage, a qualitative research into a focal group formed by Spanish students was carried out in order to find out their likes and preferences. The result was a website with an animated interface and bright colors, and which is inspired on pirates. Users are identified with a captain who fights to avoid the boarding of the ship by a crab, which symbolizes cancer (Additional file 1 and Figure 2). The contents and aims of each section of the website are summarized below:

•*Challenge chest*. This is the most important area from an educational standpoint. Every week during the academic year, students are provided with a challenge or problem to be solved, which is adapted (a) to their curriculum, (b) to their educational level and (c) to cancer prevention. The challenges were devised by Secondary Education teachers who had previously attended a training course about Health Education. The main aim of the challenges is to impact positively on students’ attitudes and self-efficacy, as well as to encourage them to follow ECAC advice. An example of a simple challenge addressed to the youngest pupils is the following: ‘*Tobacco and potato plants can be cultivated on the same field*. *If four million hectares of tobacco were replaced by potato plants*, *how many millions of tons of the latter could be harvested to reduce hunger in the world*, *having in mind that in one square meter an average of 10 kg of potatoes could be collected*? *Correct answer*: *400 million tons*’.

•*Diet Analysis*. Diet experts perform different tasks. They: (a) analyze recipes, (b) collect 24-hour food recalls sent by the students, and (c) provide online dietary advice. Its main target is to achieve a positive attitude towards preventive diets, while highlighting their many advantages.

•*Library and links*. This is an information access point, in which relevant documents and links related to epidemiology and cancer prevention are included. Students must read these references in order to solve some of the weekly challenges and to be informed about the most important events related to the program. Part of the information provided focuses on the acquisition of knowledge, while another part focuses on attitudes and social influences.

•*Games*. The computer games that appear in the program are based on the ECAC advice and aim to encourage adolescents to memorize this advice as they play. All the games provide positive feedback and emphasize the importance of putting into practice the healthy behaviors that the game suggests.

•*Videos*. They are short videos of students, imitating TV commercials. They aim to promote positive attitudes and social influence regarding the following of ECAC advice.

•*Forum and chat*. It is an open discussion forum about cancer prevention, where students are asked to give their opinion about the advantages and disadvantages of following the ECAC advice. The forum tries to promote the attitudes and social influence of their participants. This section also includes a chat room, with access control and moderation, to promote contact among its users.

The information posted by the adolescents in the discussion forum encourages the creation of a list of advantages, which is suitably prepared by experts and is sent weekly by SMS to the students. These SMS are intended (a) to serve as convincing arguments in order to stop risk behaviors and (b) to facilitate self-efficacy when implementing the cancer prevention guidelines. An example of a SMS focused on a healthy diet is the following: ‘*Don*’*t be fooled*! *The best way to be pretty on the outside is by being pretty on the inside*. *Fruits and vegetables are your best makeup*’.

### Intervention procedure

Many national and local organizations have supported Prevencanadol and have even participated in its diffusion by embedding links to our program website (http://www.alertagrumete.com) on their own institutional websites. In addition, Spanish and Mexican educational administration departments sent a massive email to all the teachers of Secondary Education, in which they explained the project and encouraged participation.

Individuals, that join voluntarily, must formalize their register in the website by providing an alias, an avatar and a private password. To complete the registry, they must fill in a compulsory online questionnaire (pre-test). After that, the computer system randomly assigns that person to one of the groups of study. Participants of the CG will have limited access to the described sections. However, those of the EG will be allowed to participate in all the proposed activities, from that very moment. At the end of each academic year, all the participants will be required to complete the same questionnaire again (post-test).

Prevencanadol is conceived as a competition: participants can obtain points in all the activities. They can receive points by different ways, such as answering the questionnaires, visiting the web for more than three minutes, watching videos, posting comments or enrolling other participants, among others. But, above all they will obtain points by solving didactic challenges. Those students and schools with the highest scores will receive a present; and they will be able to choose between different options, such as computers, mobile phones, sports equipment, books, didactic games, etc.

### Assessment of the impact of the intervention

As previously mentioned, the program also includes a pre- and post- intervention assessment. We will use them to try to determine the existing differences related to the following of the ECAC advice between (a) the very first moment in which the person is registered in the website (i.e., the beginning of the academic year) and (b) the moment in which the intervention period finishes (i.e., the end of the academic year). A complex, anonymous and self-managed questionnaire is used, and it requires approximately 20–30 minutes to be completed. It is based on another one, which has been previously validated by López et al. [29]. Notwithstanding that, it has been adapted to both young population and to online management, since it previously included graphic solutions and contact forms used to answer questions.

The obtained information allows us to design the ‛*main result variable*’, which is a synthetic indicator of the percentage of cancer risk in each individual based on his/her risk behaviors (i.e., the so called ‛global cancer behavioral risk’). This indicator is calculated by simply adding up all the risk points obtained by each person when he/she did follow the ECAC advice (Table 2); that means that a scoring of 100 shows the maximal behavioral cancer risk. These risk points are obtained from the percentage of responsibility for cancer, and according to scientific evidence, every behavior of the participant is responsible for this percentage [5-7,30,31]. To know if the individuals are following or not the advice, we will (a) apply the stages of change pointed out by Prochaska and DiClemente to each behavior and we will (b) use a questionnaire of frequency of food intake (regarding both risk foods and cancer protective foods). This questionnaire also includes information related to many variables, which according to scientific literature could modify the effect, since they influence the following (or lack of following) of the ECAC advice. These variables are: (a) *socio*-*demographic variables* (e.g., age, gender, country of residence – Spain or Mexico –, cohabitation, number of siblings, academic level, academic level of parents, weekly expenses, among others), (b) *family history of cancer* and (c) *determining factors of the A*.*S*.*E*. *Model* (i.e., social influence and self-efficacy for each behavior). Other variables which are related to the educational intervention are also controlled (e.g., SMS delivery, score obtained in the competition, number of months that the student is registered in the website, etc.).

**Table 2:** **How to calculate the indicator**: “**total cancer behavioral risk**” (**principal result variable**)

**Cancer behavioral risk**	**Points**
Smoking	35
Not eating five servings of fruit and vegetables per day	20
Eating three or more daily fatty foods	10
Quotient “frequency of protection food/risk food” ≤0.9	8
Obesity/Overweight	15/10
Excessive drinking	5
Sedentary	5
Ultraviolet radiation exposure without protection	2
Workplace exposure to carcinogens*	5
Maximum punctuation in adolescents	100^†^

*Only in adults. ^†^ 105 points in adults.

The statistical analysis will be performed through multivariate regression procedures (using the ‘Enter’ method) to control the effect of potential confounders and modifiers of the effect. Regressions will follow several steps in order to establish the percentage of variability of the result variable which is explained by the remaining variables.

### Ethical considerations

This intervention was approved by the Spanish Ministry of Health (*FISS08PI080544*) and by the Clinical Research Ethical Committee of the Principality of Asturias (Spain) (*no*. *19*/*09*). In Mexico, methodological and ethical considerations were approved by the Subsecretary of Public Education.

## Discussion

In this section we aim to discuss the main features of the methodological design of Prevencanadol:

Firstly, the project complies with the basic requirements for a parallel randomized controlled trial: (a) the study factor is artificially manipulated (online educational intervention and delivery of SMS) and (b) the study population is randomly distributed to an EG and to a CG. Notwithstanding that, this randomization is determined by the first registered student of a classroom, and consequently real classroom randomization is achieved. From this standpoint, this trial can be considered a cluster randomized controlled trial, which is a very useful design to avoid the possible contamination of the groups. However, an important limitation of the experimentation with educational interventions is that it is not possible to guarantee that participants have received the content of the intervention, as it could be done if it were a pill, for instance. But Prevencanadol has managed to reduce this limitation since it has incorporated in the statistical analysis the scoring obtained during the competition, which is an indirect indicator of the reception degree of the intervention.

Secondly, as already pointed out, the program addresses a target population between 12 and 16 years old in order to reach the highest cancer preventive efficacy, since adolescence is a vital stage in which many behaviors (that will shape the adult stage) are acquired and consolidated. Although it can be a difficult age range, prevention of risk behaviors in this population group is also possible, especially if advice is linked to the school context [32-34]. However, it is not a task that is exclusively linked to school: we should bear in mind that society and family can be important sources of behaviors [35], and therefore we also encourage the registering of adults (teachers and family) in the website http://www.alertagrumete.com. In this way, it is even possible that healthy adolescents also become instigators of some preventive behaviors among adults; that consequently achieves ascending education. Finally, regarding the diversity of participants, we should also mention that we could only have included in the program adolescents with established risk behaviors, as other authors do in their works [36], but in our opinion when working with such a young population, it is as necessary to treat existing risk behaviors as well as to prevent their presence.

Thirdly, as the intervention is both school- and web-based, it is required that adolescent participants are currently attending school and have Internet access available. In relation to the first requirement, we can affirm that about 5.4% and 13.2% of the Spanish and Mexican adolescents respectively do not regularly attend school [37], usually because they belong to disadvantaged environments. Regarding the second condition, we can conclude that in Spain almost all schools have Internet access [38], but in Mexico only less than half comply with this requirement [39]. Due to these two reasons, the intervention could be confined only to those families and schools with a higher socioeconomic level.

Fourthly, the chosen educational tools (Internet and SMS) are novel in the field of Health Education and can be easily adapted to young population. In addition, students and teachers of Secondary Education helped with the web design in order to avoid the refusal of the participating students, since it is well-known that planning this kind of interventions without bearing in mind the likes of the target population could cause them to fail even before they have been implemented [40]. However, the vertiginous evolution of this kind of technologies may have clouded the originality of this initiative, since nowadays global social networks (such as *Facebook*, *Twitter*, *Ozone*, etc.) have completely changed the way in which the web is understood. It might have even caused a certain lack of interest in the users towards other kinds of websites or minority social networks, as this one we have tried to create through http://www.alertagrumete.com.

Lastly, we would like to mention that Catalano et al. [41] published in 2012 an interesting manuscript that seems to support the approach of Prevencanadol since: (a) they acknowledge it is necessary to involve state workers (in this case, teachers) in the program; (b) these authors also suggest the identifying of both the problems and the groups in which an educational intervention is urgently needed (this is one of the aims of Prevencanadol, which we have highlighted above); and specially (c) they highlight that it is necessary to prove the efficacy of these initiatives in lower-middle income countries, as well as to find factors in these countries that can be different to those found in richer countries (and that is exactly what this program has done in Mexico).

To sum up, Prevencanadol could be considered groundbreaking, since it (a) aims to link family and school environments, (b) combines the use of the Internet and the delivery of SMS, (c) considers psychosocial models which are supported internationally, (d) intervenes in six behaviors simultaneously, and (e) compares the efficacy of the intervention in a country with a high income level (Spain) with that obtained in another one with a middle income level (Mexico).

## Abbreviations

ECAC: European Code Against Cancer; SMS: Short Text Message/Short Message Service; EG: Experimental group; CG: Control group.

## Competing interests

The authors declare no financial or non-financial competing interest.

## Authors’ contributions

All authors have made an important intellectual contribution to this project. In summary, LML is the principal researcher and has overall responsibility for the trial. LML and LA conceived and designed the whole project, even the online tool. LA also wrote the preliminary version of the manuscript and performed statistical analysis. DVMO and LS provided design consultation and follow-up of the quality intervention. F-OG gave expert drafting advice of the website contents. Specifically with respect to this paper, all authors contributed to the definitive writing of the study protocol and approved the final manuscript.

## Pre-publication history

The pre-publication history for this paper can be accessed here:

http://www.biomedcentral.com/1471-2458/13/357/prepub

## Supplementary Material

Additional file 1 Website navigation experience. Click here for file
